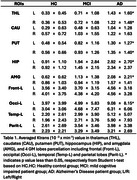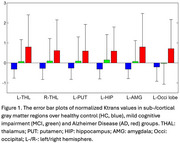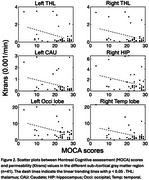# Blood Brain Barrier permeability change in clinical stages of Alzheimer disease

**DOI:** 10.1002/alz70855_105144

**Published:** 2025-12-24

**Authors:** Wanyong Shin, James B Leverenz, Mark J Lowe, Jagan A. Pillai

**Affiliations:** ^1^ Cleveland Clinic, Cleveland, OH, USA; ^2^ Cleveland Clinic Lou Ruvo Center for Brain Health, Cleveland, OH, USA; ^3^ Cleveland Clinic Lou Ruvo Center for Brain Health, Las Vegas, NV, USA

## Abstract

**Background:**

Inflammation and altered neurovascular dysfunction have been noted as features of Alzheimer's Disease (AD) pathophysiology ^1‐3^. In this context understanding nature and degree of disruption of brain blood barrier (BBB) is important in AD studies ^4‐6^, In this regard, brain region specific changes in well characterized AD subjects at distinct clinical stages of AD is yet to be well characterized. We therefore aimed to investigate the brain regional changes in BBB permeability in different clinical stages of AD versus healthy controls (HC).

**Method:**

41 subjects (70.2 ± 3.5 YO, F=26) were scanned at 3T under IRB consent. There were 6 AD dementia, 16 mild cognitive impairment from AD (MCI) and 19 HC based on clinical dementia rating scale, Montreal Cognitive assessment (MOCA) and positive AD biomarkers. Dynamic contrast enhanced (DCE) imaging was acquired with 3d GRE scans. Permeability (Ktrans) values were calculated with Patlak model ^7^ in subcortical gray matter (GM) and GM lobe parcellation ^8^.

**Result:**

We found the significant differences of averaged Ktrans values reflecting larger BBB permeability in thalamus, caudate, putamen, amygdala, hippocampus and occipital regions in AD than HC group. (see Table 1 and Figure 1). We find that the significantly negative correlation between Ktrans and MOCA scores (*p* < 0.05), as shown in Figure 2.

**Conclusion:**

We find that increased permeability in specific sub‐/cortical GM regions were more prominent in AD dementia than MCI and was significantly different from HC. These changes also linearly tracked severity of cognitive decline.

References: 1. Jellinger KA. J Neural Transm (Vienna). 2020; 2. Knopman DS, et al. J Neuropathol Exp Neurol. 2003; 3. Price JL, et al. Neurobiol Aging. 2009; 4. Fiala M, et al. Eur J Clin Invest. 2002; 5. Pillai JA, et al. Ann Clin Transl Neurol. 2019; 6. Sweeney MD, et al. Nat Rev Neurol. 2018; 7. Patlak CS, et al. J Cereb Blood Flow Metab. 1983; 8. Fischl B. Neuroimage. 2012; 9. Montagne A, et al. Neuron. 2015; 10. Nation DA, et al. Nature Medicine. 2019; 11. Sweeney MD, et al. Alzheimers Dement. 2019; 12. van de Haar HJ, et al. Radiology. 2016;